# Inventory of vascular plants and conservation-related taxa in pure *Quercus mongolica* stands in Gangwon-do, South Korea

**DOI:** 10.3897/BDJ.14.e206902

**Published:** 2026-09-18

**Authors:** Sung Je Lee, Yong-Ki Kim, Yong-Sik Hong, Kwang jin Cho

**Affiliations:** 1 Ecological Restoration Team, National Institute of Ecology, Seocheon, Republic of Korea Ecological Restoration Team, National Institute of Ecology Seocheon Republic of Korea; 2 Ecoinformatics & Control Institute, Incheon, Republic of Korea Ecoinformatics & Control Institute Incheon Republic of Korea; 3 Department of Biological Sciences, Kongju National University, Gongju, Republic of Korea Department of Biological Sciences, Kongju National University Gongju Republic of Korea

**Keywords:** conservation-related taxa, floristic inventory, floristic target plant taxa, National Ecosystem Survey, occurrence characteristics, pure stands

## Abstract

Pure *Quercus mongolica* stands are a major component of temperate deciduous broad-leaved forests in South Korea and provide important habitats for vascular plant diversity. Although *Q. mongolica* forests have been widely studied in terms of community structure, succession and relationships with environmental factors, large-scale plot-based floristic information focused specifically on stands in which *Q. mongolica* dominates the canopy layer remains limited. This study aimed to document the vascular flora of pure *Q. mongolica* stands in Gangwon-do and to provide baseline information for floristic diversity assessment, long-term monitoring and conservation management using plot-level occurrence data.

Field surveys were conducted from 2019 to 2023 across 1,098 plots established in representative pure *Q. mongolica* stands. For each plot, vascular plant occurrence data were compiled and selected environmental characteristics of the plots were summarised. The identified taxa were classified according to taxonomic rank and conservation- and management-related categories, including Korean endemic plant taxa, endangered wild plant taxa, Red List plant taxa, floristic target plant taxa, alien plant taxa and ecosystem-disturbing plant taxa.

A total of 594 vascular plant taxa were recorded, comprising 103 families, 288 genera, 539 species, 45 varieties, seven subspecies and three forms. The dataset included 20,906 plot-level occurrence records. The families with the highest numbers of taxa were Asteraceae, Rosaceae, Liliaceae, Ranunculaceae, Poaceae and Fabaceae. The number of taxa per plot ranged from 5 to 55, indicating variation in local taxon richness amongst plots of pure *Q. mongolica* stands. Conservation- and management-related taxa included 32 Korean endemic taxa, one Class II endangered wild plant taxon, 21 Korean Red List plant taxa, 172 floristic target plant taxa, seven alien plant taxa and two ecosystem-disturbing plant taxa. The floristic target plant taxa consisted of 42 Grade I, 44 Grade II, 57 Grade III, 24 Grade IV and 5 Grade V taxa. In addition, 85 taxa listed on the IUCN Red List were identified.

These results show that pure *Q. mongolica* stands in Gangwon-do contain diverse vascular plant assemblages and may serve as habitats for taxa of conservation and phytogeographical interest. The plot-based floristic inventory presented here provides baseline information for biodiversity assessment, long-term monitoring and conservation management of *Q. mongolica*-dominated temperate deciduous broad-leaved forests in Korea. By linking plot-level occurrence records with environmental metadata, the dataset can support future comparisons of floristic composition amongst *Q. mongolica* forests.

## Introduction

Pure *Quercus mongolica* stands are an important component of temperate deciduous broad-leaved forests in Korea. *Quercus mongolica* Fisch. ex Ledeb. is widely distributed throughout the Korean Peninsula and is recognised as one of the major oak species forming broad-leaved forest communities. Previous Korean studies have described *Q. mongolica* forests as a representative forest type in Korea and have examined their community structure in relation to forest succession, ecological restoration and climatic conditions (Jang et al. 1997; Lee and Song 2011; Lim and Kim 2015; Yu et al. 2024).

The ecological importance of *Q. mongolica* forests is closely related to their broad distribution, ecological adaptability and role in forest development. Studies of Korean forest vegetation have reported that *Q. mongolica* communities are widely distributed in mountainous landscapes and are associated with elevation, slope, topography and climatic conditions (Yun et al. 2010; Um 2014; Song and Yun 2019; Yu et al. 2024). Studies in Gangwon-do and the Baekdudaegan region have also shown that *Q. mongolica* forests form distinct vegetation units and occur as major forest communities on mountain ridges, slopes and natural deciduous broad-leaved forest landscapes (Jang et al. 1997; Cheon et al. 2014; Lee et al. 2014; Song and Yun 2019).

Outside Korea, *Q. mongolica* has also been treated as an important component of temperate forests in Northeast Asia. In north-eastern China and across its broader East Asian range, studies have examined changes in green-up dates and potentially suitable habitats under climate change (Fan et al. 2015; Liu et al. 2024; Kaviriri et al. 2025). In Japan, phylogeographic patterns and phytosociological relationships of summer-green broad-leaved forests have been investigated with a focus on *Q. mongolica* var*. crispula* or *Q. crispula* (Okaura et al. 2007; Shitara et al. 2024). These studies were not used as criteria for the taxonomic treatment or conservation categories in the present study, but as background literature showing that *Q. mongolica* forests can be considered within a broader Northeast Asian temperate forest context.

Research on *Q. mongolica* forests in Korea has focused on vegetation structure, community classification, succession and restoration models. For example, phytosociological and TWINSPAN-based studies have classified *Q. mongolica* forests in Gangwon-do into several community types and have demonstrated the usefulness of combining traditional phytosociological approaches with numerical classification (Jang et al. 1997). Other studies have analysed community structure, importance values, species correlations or interspecific associations and diameter-class distributions in *Q. mongolica* forests of the Baekdudaegan and other mountainous regions (Cheon et al. 2014; Lee et al. 2014; Yu et al. 2024). In studies of *Q. mongolica* communities, associated woody taxa such as *Acer pseudosieboldianum*, *Tilia amurensis*, *Fraxinus rhynchophylla* and *Rhododendron schlippenbachii* have been recorded as important understorey components (Lee and Song 2011; Yu et al. 2024).

Despite this body of domestic and international research, previous studies have generally focused on community structure, stand dynamics, diameter-class distribution, restoration models, growth, climatic responses or potential distribution modelling. In contrast, large-scale floristic inventory studies specifically targeting vascular plants occurring in pure *Q. mongolica* stands remain limited. Floristic inventories provide taxonomic and ecological baseline information for assessing plant diversity, detecting conservation-related taxa, evaluating alien plant occurrence and developing long-term monitoring strategies.

A floristic inventory becomes more reusable and comparable when it is compiled not merely as a species list, but together with plot-level occurrence data and plot metadata. Internationally, plot-based vegetation data have been used as key data sources for analysing spatial variation in plant communities, relationships with environmental gradients, long-term change and broad-scale biodiversity patterns (Dengler et al. 2011; Chytrý et al. 2016; Bruelheide et al. 2019). In Korea, floristic inventory studies, focusing on specific habitats such as forest wetlands and algific talus slopes, have presented vascular floras together with conservation-related taxa (Lee et al. 2022; Lee et al. 2023).

Gangwon-do contains extensive mountainous terrain, relatively high-elevation forest landscapes and representative temperate deciduous broad-leaved forest ecosystems, making it an important region for *Q. mongolica* forests in South Korea. In this study, pure *Q. mongolica* stands were defined as those in which *Q. mongolica* dominated the canopy layer. This definition distinguishes the target forest type from mixed forests or general forest stands in which *Q. mongolica* occurs only as one of several canopy tree species.

The objectives of this study were therefore to: (1) document the vascular plant taxa recorded in pure *Q. mongolica* stands in Gangwon-do; (2) summarise the taxonomic composition of the flora and plot-level occurrence patterns; (3) identify conservation- and management-related taxa, including Korean endemic plant taxa, endangered wild plant taxa, Red List plant taxa, floristic target plant taxa, alien plant taxa and ecosystem-disturbing plant taxa; and (4) compile plot-based occurrence data and summarise environmental characteristics as baseline floristic information for biodiversity assessment, long-term monitoring and conservation management of *Q. mongolica* forests. By integrating plot-level occurrence records from 1,098 plots, this study focuses on providing a reusable floristic reference dataset for pure *Q. mongolica* stands in Gangwon-do.

## Material and Methods

### Study area

This study was conducted in Gangwon-do, a mountainous region that includes extensive temperate deciduous broad-leaved forests and areas in which *Q. mongolica* forests are distributed.

**Figure 1.**
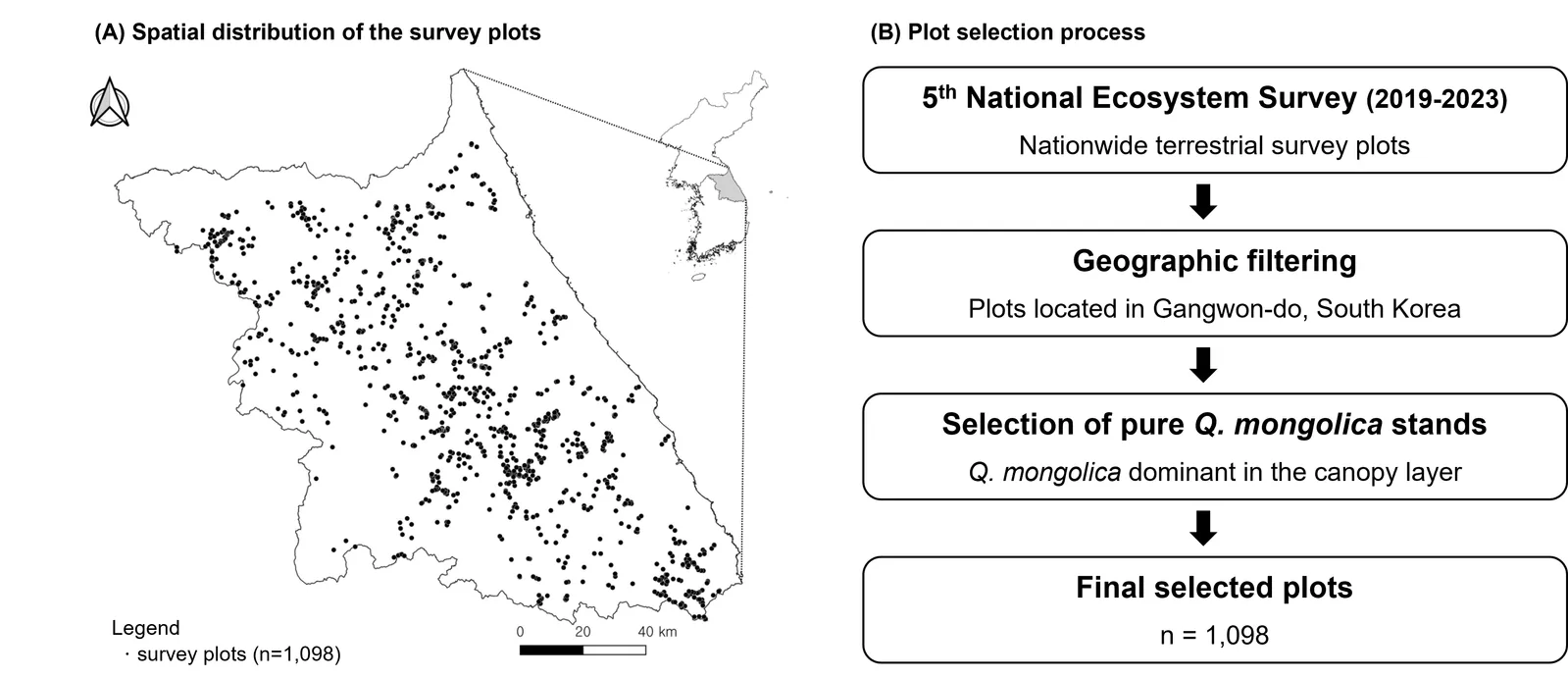
Spatial distribution and selection process of survey plots in pure *Q. mongolica* stands in Gangwon-do, South Korea: (A) Spatial distribution of the 1,098 selected survey plots; (B) Flowchart of the plot selection process, based on geographic location and canopy-layer dominance of *Q. mongolica*.

In this study, the term “pure *Q. mongolica* stands” was used as an operational definition for delimiting the dataset. The term referred to plots from the 5^th^ National Ecosystem Survey in which *Q. mongolica* was recorded as the dominant species in the canopy layer in the original field survey records. It does not imply monospecific stands or the absence of other woody species in the canopy, subcanopy or shrub layers. This definition was used to distinguish the target stands from mixed forests or general forest stands in which *Q. mongolica* occurs only as one component of the tree layer. A total of 1,098 plots satisfying this criterion were included in the final analysis and the spatial distribution of the plots is shown in Fig. 1.

### Field surveys and floristic data

Floristic data were obtained from field investigations conducted from 2019 to 2023 as part of the 5^th^ National Ecosystem Survey (National Institute of Ecology 2019). From the 5^th^ National Ecosystem Survey, plots meeting the operational definition described above were selected and all vascular plant taxa recorded in each plot were compiled as plot-level occurrence data. For analysis, the original plot-level occurrence data were summaried to calculate taxon occurrence frequency and plot-level taxon richness, with a detailed occurrence record dataset also provided through GBIF.

The final floristic dataset consisted of 1,098 plots and 20,906 plot-level occurrence records.

The number of taxa recorded in each plot was calculated as plot-level taxon richness. Taxonomic composition was summarised at the levels of family, genus, species, variety, subspecies and form. The families with the highest numbers of taxa and the most frequently recorded taxa were identified on the basis of the number of taxa per family and the number of plots in which each taxon occurred, respectively.

### Environmental variables and data sources

Environmental variables were summarised to describe the survey scope and environmental setting of the surveyed pure *Q. mongolica* stands, rather than to serve as a main analytical axis of this floristic inventory study.

Environmental data were compiled from field survey records and spatial datasets. Field-based variables used in the internal compilation included plot size, topography, soil type, moisture condition, lithology and field-recorded soil texture; selected numerical and categorical variables are summarised in the Results, Table 7 and Supplementary material 3. Soil-related spatial variables, including deep soil texture, soil drainage class and effective soil depth, were obtained from spatial datasets provided by the National Academy of Agricultural Science through World Spatial Information Download (National Academy of Agricultural Science 2023a; National Academy of Agricultural Science 2023b; National Academy of Agricultural Science 2024). Elevation and slope were extracted from NASADEM 30 m data (NASA JPL 2021). Vegetation-climatic zone information followed the classification of Cho et al. (2020).

To ensure spatial consistency amongst plots, NASADEM-derived elevation and slope were used as representative variables instead of the values recorded on field survey sheets. These variables are referred to as NASADEM_GlobalDEM and Slope_NASADEM30m, respectively.

### Taxonomic treatment

All recorded vascular plant taxa were standardised at the levels of family, genus, species, variety, subspecies and form. Each taxon was treated as an operational floristic unit for summarising taxon richness and plot-level occurrence patterns. Scientific and Korean names were based primarily on the National List of Korea dataset from the National Institute of Biological Resources and public data obtained through the Public Data Portal (data.go.kr). Some taxa absent from domestic databases or requiring taxonomic review, such as Veratrum versicolor f. viride, were supplemented through the Korea Biodiversity Information System of the Korea National Arboretum. Scientific names that could not be definitively confirmed in domestic databases were checked and verified using the international databases IPNI (International Plant Names Index) and POWO (Plants of the World Online) (National Institute of Biological Resources 2025a; Korea National Arboretum 2026; IPNI 2026; POWO 2026).

### Classification of conservation- and management-related taxa

The recorded taxa were classified into conservation- and management-related categories to describe taxa of conservation or management interest recorded in pure *Q. mongolica* stands. These categories included Korean endemic plant taxa, endangered wild plant taxa, Red List plant taxa, floristic target plant taxa, alien plant taxa and ecosystem-disturbing plant taxa.

Korean endemic plant taxa were identified using the national endemic species reference provided by the National Institute of Biological Resources (National Institute of Biological Resources 2025b).

Endangered wild plant taxa were classified according to the national list under the Wildlife Protection and Management Act of South Korea (Ministry of Climate, Energy and Environment 2026). Korean Red List plant taxa were classified using the national Red List reference for vascular plants (National Institute of Biological Resources 2021). Floristic target plant taxa were classified into Grades I–V following national references for floristic target plant taxa (National Institute of Ecology 2018). Alien plant taxa and ecosystem-disturbing plant taxa were identified using the national alien species list and the official designation notice for ecosystem-disturbing species, respectively (National Institute of Ecology 2025; Ministry of Environment 2025). IUCN Red List plant taxa were classified using a list of 481 vascular plant species extracted from the full IUCN Red List by applying the taxonomic filter for vascular plants and the IUCN land region filter for South Korea (IUCN 2026).

Conservation- and management-related categories were not combined into a single integrated rank, but were summarised separately according to the purpose of each legal or reference list. For example, alien plant taxa and ecosystem-disturbing plant taxa were treated as independent categories because they are based on different sources and designation purposes. This classification clarifies the conservation or management context in which each taxon is relevant.

### Data analysis

All analyses were conducted at the taxon and plot levels. First, the total number of vascular plant taxa was calculated and summarised by taxonomic rank. Second, the number of taxa in each family was calculated to identify major taxon-rich families in the flora. Third, the number of taxa recorded in each plot was counted to obtain plot-level taxon richness. Fourth, the occurrence frequency of each taxon was calculated on the basis of the number of plots in which the taxon was recorded.

For conservation-related taxa, the number of taxa in each category was calculated. Korean endemic plant taxa, endangered wild plant taxa, Red List plant taxa, floristic target plant taxa, alien plant taxa and ecosystem-disturbing plant taxa were summarised separately. Floristic target plant taxa were further summarised by grade and Red List plant taxa were summarised by threat category.

Environmental variables were summarised using descriptive statistics to characterise the ecological setting of the plots. For numerical variables such as elevation, slope, plot width and length, minimum, maximum, mean and median values were calculated. For categorical variables, such as topography, moisture condition, soil type and vegetation-climatic zone, the number of plots in each category was counted. These environmental summaries were used to describe the survey scope and plot metadata, rather than to infer causal relationships between environmental variables and species composition.

## Data resources

**Dataset Title**: nes5-qm-vepl-gw (Plot-level vascular plant occurrences in pure *Q. mongolica* stands in Gangwon-do, South Korea)

**Data Repository**: Global Biodiversity Information Facility (GBIF)

**Identifier (DOI)**: https://doi.org/10.15468/78chgh

**License**: CC-BY-NC-4.0

**Description**:

The floristic dataset constructed in this study consists of plot-level occurrence records of vascular plants recorded in pure *Q. mongolica* stands in Gangwon-do, as operationally defined in this study. The dataset was generated from the 5^th^ National Ecosystem Survey conducted from 2019 to 2023 and includes 1,098 plots, 594 vascular plant taxa and 20,906 plot-level occurrence records.

The supplementary materials contain three data tables: occurrence frequency of all taxa, conservation- and management-related taxa and a categorical summary of environmental variables. In addition, the occurrence record dataset of this study is registered and publicly available in the Global Biodiversity Information Facility (GBIF).

## Results

### Taxonomic composition of the vascular flora

A total of 594 vascular plant taxa were recorded in 1,098 plots in pure *Q. mongolica* stands in Gangwon-do. These taxa comprised 103 families, 288 genera, 539 species, 45 varieties, seven subspecies and three forms. The floristic dataset included 20,906 plot-level occurrence records. The taxonomic composition is summarised in Table 1.

Species-level taxa accounted for the majority of the recorded flora, with 539 species. Infraspecific taxa consisted of 45 varieties, seven subspecies and three forms.

### Composition of major taxon-rich families

Asteraceae had the highest number of taxa (52), followed by Rosaceae (44), Liliaceae (37), Ranunculaceae (29), Poaceae (21) and Fabaceae (20). Other major families included Lamiaceae, Apiaceae, Betulaceae, Violaceae and Dryopteridaceae. The numbers of taxa in major families are presented in Table 2.

### Plot-level taxon richness and occurrence records

**Figure 2.**
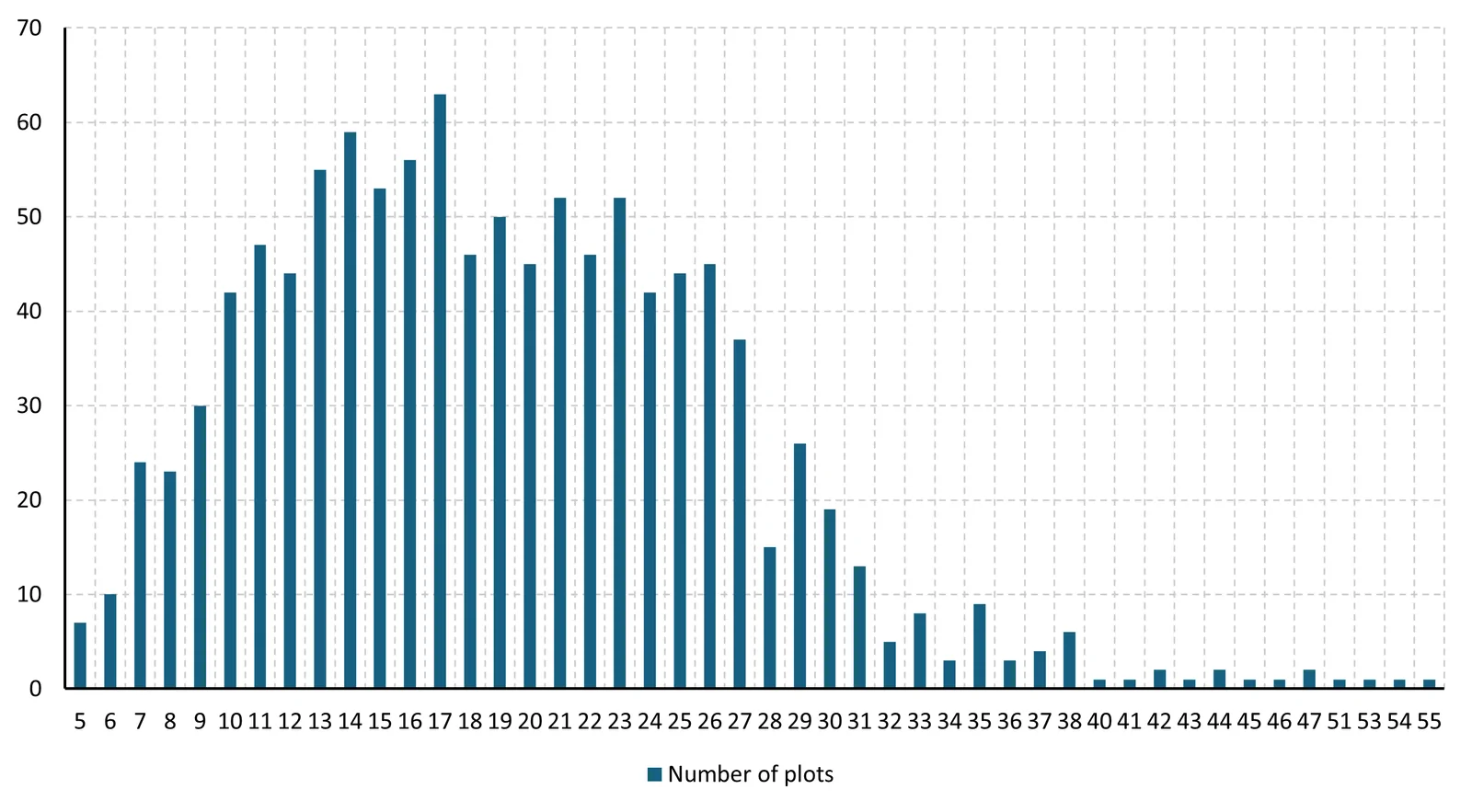
Distribution of plot-level vascular plant taxon richness in pure *Q. mongolica* stands.

The number of vascular plant taxa per plot ranged from 5 to 55. Mean plot-level taxon richness was 19.04 taxa per plot and the median was 18 taxa. The first and third quartiles were 13 and 24 taxa, respectively, indicating that the middle 50% of plots contained approximately 13–24 taxa. Summary statistics are presented in Table 3 and the distribution of plot-level taxon richness is shown in Fig. 2.

Variation in plot-level taxon richness indicates that local floristic composition differed amongst plots, even though all plots were defined as pure stands dominated by *Q. mongolica* in the canopy layer. Such variation may reflect differences in microtopography, stand structure, disturbance history, understorey development and local environmental conditions. However, because this study was designed as a floristic inventory, the environmental drivers of plot-level taxon richness were not analysed as a main objective.

### Most frequently recorded taxa

*Quercus mongolica* was recorded in all 1,098 plots, confirming that all plots belonged to the target forest type. The most frequently recorded associated taxa included *Lindera obtusiloba*, *Acer pseudosieboldianum*, *Fraxinus rhynchophylla*, *Carex siderosticta*, *Rhododendron schlippenbachii*, *Lespedeza maximowiczii*, *Symplocos sawafutagi*, *Ainsliaea acerifolia* and *Rhododendron mucronulatum*. The most frequent taxa are summarised in Table 4 and Suppl. material 1.

Woody taxa such as *Lindera obtusiloba*, *Acer pseudosieboldianum*, *Fraxinus rhynchophylla*, *Rhododendron schlippenbachii* and *Rhododendron mucronulatum* were repeatedly recorded across many plots and herbaceous taxa, such as *Carex siderosticta* and *Ainsliaea acerifolia*, also showed high occurrence frequencies. These results indicate that the vascular flora of pure *Q. mongolica* stands includes not only the canopy-dominant species, but also understorey woody and forest-floor herbaceous taxa.

### Conservation-related taxa

A total of 32 Korean endemic taxa were recorded in plots of pure *Q. mongolica* stands. One taxon was classified as an endangered wild plant of Class II. A total of 21 Korean Red List plant taxa were recorded, consisting of 11 Least Concern (LC), four Endangered (EN), three Near Threatened (NT), two Vulnerable (VU) and one Data Deficient (DD) taxa. A total of 85 IUCN Red List plant taxa were recorded, consisting of two Data Deficient (DD), four Endangered (EN), 76 Least Concern (LC), two Near Threatened (NT) and one Vulnerable (VU) taxa. The conservation-related taxa and composition of Red List categories are presented in Table 5 and Suppl. material 2.

Floristic target plant taxa were particularly numerous. A total of 172 floristic target plant taxa were recorded, consisting of 42 Grade I, 44 Grade II, 57 Grade III, 24 Grade IV and five Grade V taxa. Grade III taxa were the most numerous, followed by Grade I and Grade II taxa. The grade-level composition is summarised in Table 5.

These conservation-related taxa provide occurrence evidence for conservation and phytogeographical elements within pure *Q. mongolica* stands in Gangwon-do. In particular, the occurrence of Grade IV and Grade V floristic target plant taxa shows that this forest type may provide occurrence sites for some taxa with restricted distributions or phytogeographical significance.

### Alien plant taxa and ecosystem-disturbing plant taxa

A total of seven alien plant taxa and two ecosystem-disturbing plant taxa were recorded in the plots. As this study was not designed to quantitatively assess invasion intensity, these results should be interpreted as evidence of the presence of management-relevant taxa in pure *Q. mongolica* stands and the need for future monitoring. Category-level counts are presented in Table 6.

### Environmental characteristics of the plots

The 1,098 plots covered a broad environmental range within Gangwon-do. According to NASADEM-derived terrain variables, elevation ranged from 24 m to 1,450 m, with a mean of 732.6 m and a median of 747.5 m. NASADEM-derived slope ranged from 0.3 to 45.5°, with a mean of 19.3° and a median of 19.4°. Plot width ranged from 10 m to 25 m, with a mean of 13.1 m and a median of 11 m, while plot length ranged from 5 m to 25 m, with a mean of 13.1 m and a median of 11 m. Numerical summaries of the main environmental and plot-size characteristics are presented in Table 7. Categorical summaries of environmental characteristics are provided in Suppl. material 3.

Most plots were located on upper and middle slopes. Upper slopes accounted for the largest number of plots (513 plots), followed by middle slopes (303), lower slopes (127), ridges (101), valleys (47) and summits (7). Moisture conditions were predominantly mesic, with 627 plots classified as mesic, 262 as slightly moist and 190 as slightly dry. Excessively wet and excessively dry conditions were uncommon, occurring in 18 plots and one plot, respectively.

Vegetation-climatic zone information indicated that most plots were located in the northern temperate deciduous broad-leaved forest zone. This zone included 969 plots, followed by the central temperate deciduous broad-leaved forest zone with 95 plots. The northern temperate mixed coniferous and broad-leaved forest zone, southern temperate mixed evergreen-deciduous broad-leaved forest zone and southern temperate deciduous broad-leaved forest zone included 19, 14 and one plots, respectively.

Brown forest soil was the dominant soil type, occurring in 1,024 plots, followed by red-yellow forest soil in 36 plots, dark red forest soil in 34 plots and grey-brown forest soil in four plots. Lithology was mainly igneous rock, which occurred in 562 plots, followed by metamorphic rock in 469 plots and sedimentary rock in 67 plots. Surface soil texture was dominated by loam, with 981 plots, whereas sandy and clayey/sandy soils occurred in 59 and 58 plots, respectively. Deep soil texture was mainly sandy loam, occurring in 947 plots, followed by clay loam in 135 plots, other soil textures in nine plots, clayey soil in four plots, sandy soil in two plots and gravelly soil in one plot.

Drainage was generally good. Most plots were classified as very well drained (859 plots) or well drained (228 plots), whereas the other and moderately well-drained classes occurred in nine and two plots, respectively. The most frequent effective soil depth classes were 50–100 cm and 20–50 cm, recorded in 437 and 435 plots, respectively. Shallow soils less than 20 cm deep occurred in 211 plots, whereas soils deeper than 100 cm were recorded in only six plots. Nine plots were classified as other.

These results indicate that the surveyed pure *Q. mongolica* stands were distributed mainly in the northern temperate deciduous broad-leaved forest zone of Gangwon-do, particularly on upper and middle mountain slopes under mesic to slightly moist conditions. The plots were also mainly associated with brown forest soils, igneous or metamorphic bedrock, loamy surface soils, sandy loam subsoils, well-drained to very well-drained conditions and effective soil depths of 20–100 cm. Therefore, the surveyed plots were mainly located on mountain slopes with relatively good drainage and intermediate effective soil depth and these site conditions represent the main environmental characteristics identified in this study.

## Discussion

### Floristic composition of pure *Q. mongolica* stands

This study provides a large-scale plot-based floristic inventory of pure *Q. mongolica* stands in Gangwon-do. A total of 594 vascular plant taxa were recorded in 1,098 plots, yielding 20,906 plot-level occurrence records. These results show that pure *Q. mongolica* stands, as operationally defined by the canopy-layer dominance of *Q. mongolica* in the original field survey records, contain a diverse vascular flora composed of woody plants, forest herbs, sedges, ferns and infraspecific taxa.

Previous studies of *Q. mongolica* forests in Korea have focused on vegetation structure, community classification, succession, diameter-class distribution or restoration models (Jang et al. 1997; Lee and Song 2011; Lee et al. 2014; Yu et al. 2024). By focusing on the floristic composition of pure *Q. mongolica* stands and integrating plot-level occurrence information across many plots, this study provides an occurrence-based floristic reference that complements these vegetation-structure studies.

In the international literature, studies from north-eastern China and across the broader East Asian range have examined phenological responses, climatic effects and changes in potentially suitable habitats and ecological niches of *Q. mongolica* under climate change (Fan et al. 2015; Liu et al. 2024; Kaviriri et al. 2025), whereas Japanese studies have addressed phytogeographical and phytosociological relationships with Northeast Asian summer-green broad-leaved forests, mainly in relation to *Q. mongolica var. crispula* or *Q. crispula* (Okaura et al. 2007; Shitara et al. 2024). Although the present dataset does not share the same analytical framework as those international studies, it can provide comparative floristic reference data on vascular plant occurrence in pure *Q. mongolica* stands in Gangwon-do for broader studies of Northeast Asian oak forests.

The high occurrence frequencies of *Lindera obtusiloba*, *Acer pseudosieboldianum*, *Fraxinus rhynchophylla*, *Carex siderosticta*, *Rhododendron schlippenbachii*, *Ainsliaea acerifolia*, *Lespedeza maximowiczii, Symplocos sawafutagi* and *Rhododendron mucronulatum* indicate that these taxa were repeatedly recorded in pure *Q. mongolica* stands in Gangwon-do. This pattern is broadly consistent with previous studies reporting *Acer pseudosieboldianum*, *Rhododendron schlippenbachii*, *Tilia amurensis*, *Fraxinus rhynchophylla* and other deciduous broad-leaved taxa as important associated taxa in *Q. mongolica* communities (Suh and Lee 1998; Lee and Song 2011; Lee et al. 2014; Yu et al. 2024). However, the occurrence frequencies in this study are based on plot-level presence data rather than cover or importance values and, therefore, should not be interpreted as measures of dominance or relative ecological importance within communities.

### Complementarity to studies of *Q. mongolica* forest structure

The results of this study are broadly consistent with previous studies that describe *Q. mongolica* communities as important deciduous broad-leaved forest types in Korea. A recent study of *Q. mongolica* communities in the central region of Korea reported that *Q. mongolica* had the highest importance value in both the tree and subtree layers and suggested that these communities may maintain *Q. mongolica* dominance over the long term (Yu et al. 2024). Similarly, Lee et al. (2014) indicated, on the basis of species composition and diameter-class distributions, that *Q. mongolica* forests may persist as stable forest communities. Song and Yun (2019) showed that *Q. mongolica* community groups are related to environmental gradients in the Baekdudaegan Region.

The present study differs in its main purpose from such community-structure studies. Instead of classifying vegetation units or predicting succession using TWINSPAN, CCA or importance-value analyses, this study provides a floristic inventory and an occurrence-based reference dataset for pure *Q. mongolica* stands. Whereas community-structure studies show how dominant and associated species are organised by vegetation layer, the floristic inventory presented here describes the range of vascular plant taxa actually recorded in a particular forest type and their plot-level occurrence patterns. Therefore, this dataset should be interpreted not as a substitute for existing vegetation-classification or vegetation-structure studies, but as a complementary reference for vascular plant occurrence in pure *Q. mongolica* stands.

The focus on pure stands is also important. Previous studies have often analysed *Q. mongolica* communities together with mixed forests or compared *Q. mongolica* forests with other forest types (Lee et al. 2014; Lim and Kim 2015). By restricting the dataset to stands in which *Q. mongolica* dominates the canopy layer, this study provides a clearer floristic reference for a specific forest type. This approach reduces interpretive ambiguity caused by mixed forests and helps identify the vascular plant assemblage recorded in pure *Q. mongolica* stands.

###  Conservation- and management-related taxa in pure *Quercus mongolica* stands

The conservation-related taxa recorded in this study provide occurrence evidence for conservation and phytogeographical elements within pure *Q. mongolica* stands. Floristic target plant taxa are used as indicators for identifying regionally or nationally important floristic elements and the grade-level occurrence information obtained in this study can support future interpretations of the phytogeographical characteristics of this forest type (National Institute of Ecology 2018).

The occurrence of Red List plant taxa and an endangered wild plant taxon indicates that pure *Q. mongolica* stands include occurrence sites for some taxa of conservation concern. However, because this study did not quantitatively assess population size or growth status, these results should be interpreted as occurrence information based on national and international reference lists, rather than as a new assessment of the conservation status of each taxon. Such occurrence information may help identify taxa that should be prioritised for confirmation in future repeated surveys and habitat management.

The records of seven alien plant taxa and two ecosystem-disturbing plant taxa show that some management-relevant taxa occur even within the surveyed pure *Q. mongolica* stands. Although the present data do not allow direct comparison of alien plant invasion intensity with other habitats, the occurrence of ecosystem-disturbing plant taxa suggests that continued monitoring may be needed. Previous studies have also emphasised the need for continued monitoring and management of alien plants because their distribution and spread can vary with environmental conditions and human disturbance (Jung et al. 2017; Kang et al. 2020). Monitoring of alien plant taxa and ecosystem-disturbing plant taxa can be treated as a component of the long-term management of pure *Q. mongolica* stands. The plot-level occurrence data provided in this study can be used to identify plots in which alien and ecosystem-disturbing plant taxa were recorded and may, therefore, provide a basis for targeted follow-up surveys and long-term monitoring.

### Reuse potential for monitoring and forest management

This study contributes to the development of a reusable floristic dataset in three ways. First, it provides a taxonomic summary of vascular plants recorded in pure *Q. mongolica* stands. Second, it presents plot-level occurrence records that can be reused for future biodiversity assessments and monitoring. Third, it summarises conservation- and management-related taxa, including endemic plant taxa, Red List plant taxa, floristic target plant taxa, alien plant taxa and ecosystem-disturbing plant taxa, as separate categories. Internationally, plot-based vegetation data have been used as core data sources for large vegetation databases and broad-scale biodiversity analyses and the plot-level occurrence data from this study can likewise be reused for future data integration and comparative research (Dengler et al. 2011; Chytrý et al. 2016; Bruelheide et al. 2019).

In this study, environmental variables were used mainly to describe the survey scope and environmental setting of the plots, rather than to infer causal relationships between plant occurrence and environmental gradients. This was an intentional choice because the primary objective was to provide a floristic inventory based on plot-level occurrence records. If plot-level environmental metadata are made available together with the occurrence records, these data could provide input for future studies of species distributions, habitat preferences, plot-level taxon richness and floristic change.

### Data value for habitat-based floristic studies

The target habitat in this study was defined as pure *Q. mongolica* stands, as operationally delimited by the canopy-layer dominance of *Q. mongolica* in the original field survey records. This definition provides forest-type-specific occurrence information that cannot be readily obtained from general regional floras. For example, the dataset can be used to identify which vascular plant taxa are repeatedly recorded in pure *Q. mongolica* stands and which conservation- and management-related taxa occur within this forest type.

The plot-level structure of the dataset also increases its value. A simple species list can show which taxa occur in a region, but plot-level occurrence records indicate how widely each taxon is distributed within the target habitat. The high occurrence frequencies of species, such as *Lindera obtusiloba*, *Acer pseudosieboldianum*, *Fraxinus rhynchophylla*, *Carex siderosticta* and *Rhododendron schlippenbachii*, suggest that these taxa were frequently recorded in pure *Q. mongolica* stands in Gangwon-do. Conversely, rare or low-frequency taxa may be useful as candidates for further confirmation in future surveys, especially when they are also included in conservation- or management-related categories.

This study, therefore, contributes not only to records of forest flora in Gangwon-do, but also to the accumulation of biodiversity data for *Q. mongolica*-dominated temperate deciduous broad-leaved forests in Korea. By presenting the total flora together with conservation- and management-related categories, this study provides occurrence-based information that can be used in taxonomy, biodiversity monitoring, forest conservation and ecological management.

### Limitations and future research

This study has several limitations. First, the analyses were based mainly on presence-absence data. Therefore, abundance, cover, dominance and vertical-layer structure of each taxon were not evaluated. For this reason, the results should be interpreted as a floristic and occurrence-based inventory rather than a complete vegetation-structure analysis.

Second, environmental variables were summarised descriptively and were not used to model species composition or plot-level taxon richness. Future studies could examine relationships between vascular plant occurrence and environmental gradients by combining plot-level occurrence matrices with environmental metadata. Such analyses would be useful for understanding how elevation, slope, moisture conditions, soil characteristics and climatic conditions are related to vascular plant richness, composition and the occurrence of conservation-related taxa (Lee et al. 2013; Woo et al. 2025; Park and Cheon 2025).

Third, this study focused on pure *Q. mongolica* stands in Gangwon-do. Therefore, the results should not be directly generalised to all *Q. mongolica* forests in Korea, especially mixed oak forests, urban *Q. mongolica* stands or forests in southern regions. To evaluate how floristic composition varies across the broader distribution range of *Q. mongolica* forests, future studies comparing different provinces, vegetation-climatic zones and pure versus mixed stands would be useful.

Finally, forest communities can be affected by climate change, pests, disturbances and management practices; therefore, repeated surveys are needed to detect temporal changes in species composition and conservation-related taxa. Studies of *Q. mongolica* in north-eastern China and across its broader East Asian range have analysed changes in green-up dates and potentially suitable habitats in relation to climate change (Fan et al. 2015; Liu et al. 2024; Kaviriri et al. 2025), suggesting the need to accumulate *Q. mongolica* forest data as reference datasets for long-term monitoring and future assessments of climate-related floristic change. However, because this study did not directly analyse climatic responses, relationships between climate change and floristic change should be examined through repeated surveys and separate analyses in the future.

## Conclusions

This study provides a large-scale plot-based floristic inventory specifically targeting pure *Q. mongolica* stands in Gangwon-do. Based on 1,098 plots surveyed from 2019 to 2023, a total of 594 vascular plant taxa were recorded, comprising 103 families, 288 genera, 539 species, 45 varieties, seven subspecies and three forms. The dataset includes 20,906 plot-level occurrence records, showing that pure *Q. mongolica* stands contain diverse vascular plants despite being operationally defined by the canopy-layer dominance of *Q. mongolica* in the original field survey records.

The results also show that pure *Q. mongolica* stands contain taxa of conservation and phytogeographical interest. The recorded flora included 32 Korean endemic taxa, one Class II endangered wild plant taxon, 21 Korean Red List plant taxa and 172 floristic target plant taxa. A total of 85 taxa were identified as IUCN Red List plant taxa. The occurrence of seven alien plant taxa and two ecosystem-disturbing plant taxa indicates that these taxa should be considered for confirmation in future repeated surveys and considered in management.

By integrating plot-level occurrence data and summarising environmental characteristics, this study provides a reusable floristic dataset for biodiversity assessment, long-term monitoring and conservation management of *Q. mongolica* forests. This floristic inventory can support regional comparisons of floristic composition, detection of future changes in species occurrence, conservation-priority assessment and habitat-based management of *Q. mongolica*-dominated temperate deciduous broad-leaved forests.

## Supplementary Material

C91E01E4-2EE0-5694-995E-CEE0AA4175D8 10.3897/BDJ.14.e206902.suppl1 Supplementary material 1 Occurrence frequency of all taxa Data type occurrences File: oo_1704882.xlsx https://binary.pensoft.net/file/1704882 Sung Je Lee; Yong-Ki Kim; Yong-Sik Hong; Kwang jin Cho

17F53E63-6A9E-52C6-8F41-F85D8066B1E5 10.3897/BDJ.14.e206902.suppl2 Supplementary material 2 Conservation- and management-related taxa Data type occurrences File: oo_1704612.xlsx https://binary.pensoft.net/file/1704612 Sung Je Lee; Yong-Ki Kim; Yong-Sik Hong; Kwang jin Cho

F941AEBE-E703-5DB9-8E5E-EE6890829242 10.3897/BDJ.14.e206902.suppl3 Supplementary material 3 Categorical summary of environmental variables Data type variables File: oo_1704606.xlsx https://binary.pensoft.net/file/1704606 Sung Je Lee; Yong-Ki Kim; Yong-Sik Hong; Kwang jin Cho

## Figures and Tables

**Table 1.** Taxonomic composition of the vascular flora recorded in pure *Q. mongolica* stands in Gangwon-do.

Category	Number
Families	103
Genera	288
Species	539
Varieties	45
Subspecies	7
Forms	3
Total taxa	594

**Table 2.** Major taxon-rich families recorded in pure *Q. mongolica* stands in Gangwon-do.

Rank	Family	Number of taxa	Percentage of total taxa (%)
1	Asteraceae	52	8.8
2	Rosaceae	44	7.4
3	Liliaceae	37	6.2
4	Ranunculaceae	29	4.9
5	Poaceae	21	3.5
6	Fabaceae	20	3.4
7	Lamiaceae	17	2.9
8	Apiaceae	16	2.7
9	Betulaceae	15	2.5
9	Violaceae	15	2.5
9	Dryopteridaceae	15	2.5

**Table 3.** Summary of taxon richness per plot in pure *Q. mongolica* stands.

Statistic	Number of taxa per plot
Minimum	5
First quartile	13
Median	18
Mean	19.04
Third quartile	24
Maximum	55

**Table 4.** Most frequently recorded taxa in pure *Q. mongolica* stands in Gangwon-do.

Rank	Taxon	Family	Plots	Occ. (%)
1	*Quercus mongolica* Fisch. ex Ledeb.	Fagaceae	1,098	100.0
2	*Lindera obtusiloba* Blume	Lauraceae	865	78.8
3	*Acer pseudosieboldianum* (Pax) Kom.	Aceraceae	698	63.6
4	*Fraxinus rhynchophylla* Hance	Oleaceae	576	52.5
5	*Carex siderosticta* Hance	Cyperaceae	568	51.7
6	*Rhododendron schlippenbachii* Maxim.	Ericaceae	485	44.2
7	*Lespedeza maximowiczii* C. K. Schneid.	Fabaceae	383	34.9
8	*Symplocos sawafutagi* Nagam.	Symplocaceae	378	34.4
9	*Ainsliaea acerifolia* Sch. Bip.	Asteraceae	377	34.3
10	*Rhododendron mucronulatum* Turcz.	Ericaceae	354	32.2

**Table 5.** Conservation-related vascular plant taxa recorded in pure *Q. mongolica* stands in Gangwon-do.

Category		Number of taxa
Korean endemic plant taxa		32
Endangered wild plant taxa, Class II		1
Korean Red List plant taxa, total		21
Korean Red List	Least Concern (LC)	11
	Endangered (EN)	4
	Near Threatened (NT)	3
	Vulnerable (VU)	2
	Data Deficient (DD)	1
IUCN Red List plant taxa, total		85
IUCN Red List	Least Concern (LC)	76
	Endangered (EN)	4
	Near Threatened (NT)	2
	Vulnerable (VU)	1
	Data Deficient (DD)	2
Floristic target plant taxa, total		172
Floristic target plant taxa	Grade I	42
	Grade II	44
	Grade III	57
	Grade IV	24
	Grade V	5

**Table 6.** Management-related alien plant categories recorded in pure *Q. mongolica* stands.

Category	Number of taxa
Alien plant taxa	7
Ecosystem-disturbing plant taxa	2

**Table 7.** Numeric summary of environmental variables.

Variable	Unit	No. of plots	Minimum	Maximum	Mean	Median	SD
Elevation	m	1098	24	1450	732.6	747.5	257.2
Slope	degree	1098	0.33	45.48	19.29	19.39	7.68
Plot width	m	1098	10	25	13.1	11	3.5
Plot length	m	1098	5	25	13.1	11	3.5

## References

[B14294315] Bruelheide Helge, Dengler Jürgen, Jiménez‐Alfaro Borja (2019). sPlot – A new tool for global vegetation analyses. Journal of Vegetation Science.

[B14292328] Cheon Kwangil, Byun Jungi, Jung Sungcheol, Sung Joohan (2014). Community structure of *Quercus
mongolica* stand in Hyangrobong Area, Baekdudaegan. Journal of Agriculture & Life Science.

[B14292338] Cho Yong-Chan, Jung Song-hie, Lee Dong-hyuk, Kim Han-gyeol, Jae-hyun Kim (2020). Forest of Korea (VI): Biogeography of Korea: Flora and vegetation.

[B14294324] Chytrý Milan, Hennekens Stephan M., Jiménez‐Alfaro Borja (2016). European Vegetation Archive (EVA): an integrated database of European vegetation plots. Applied Vegetation Science.

[B14292448] Dengler Jürgen, Jansen Florian, Glöckler Falko, Peet Robert K., De Cáceres Miquel, Chytrý Milan, Ewald Jörg, Oldeland Jens, Lopez‐Gonzalez Gabriela, Finckh Manfred, Mucina Ladislav, Rodwell John S., Schaminée Joop H. J., Spencer Nick (2011). The Global Index of Vegetation‐Plot Databases (GIVD): a new resource for vegetation science. Journal of Vegetation Science.

[B14292467] Fan Deqin, Zhu Wenquan, Zheng Zhoutao, Zhang Donghai, Pan Yaozhong, Jiang Nan, Zhou Xiafei (2015). Change in the green-up dates for *Quercus
mongolica* in Northeast China and its climate-driven mechanism from 1962 to 2012. PLOS One.

[B14292479] IPNI International Plant Names Index. The Royal Botanic Gardens, Kew, Harvard University Herbaria & Libraries and Australian National Herbarium. https://www.ipni.org/.

[B14292487] IUCN The IUCN Red List of Threatened Species. https://www.iucnredlist.org/.

[B14292495] Jang Kyu Kwan, Song Ho Kyung, Kim Seong Deog (1997). Study on classification of *Quercus
mongolica
f*orests in Kangwon - do by phytosociological method and TWINSPAN. Journal of Korean Forest Society.

[B14292540] Jung S. Y., Lee J. W., Shin H. T., Kim S. J., An J. B., Heo T. I., Chung J. M., Cho Y. C. (2017). Invasive alien plants in South Korea.

[B14292552] Kang Eun Su, Lee Soo-Rang, Oh Seung Hwan, Kim Dong-Kap, Jung Su-Young, Son Dong Chan (2020). Comprehensive review about alien plants in Korea. Korean Journal of Plant Taxonomy.

[B14292563] Kaviriri David Kombi, Liu Tianyi, Yang Ling (2025). Potential distribution and ecological niche of *Quercus
mongolica* under different climate scenarios. Plant Ecology.

[B14292572] Arboretum Korea National Korea Biodiversity Information System. https://www.nature.go.kr/.

[B14292580] Lee Chang-Bae, Chun Jung-Hwa, Um Tae-Won, Cho Hyun-Je (2013). Altitudinal patterns and determinants of plant species richness on the Baekdudaegan Mountains, South Korea: common versus rare species. Journal of Ecology and Environment.

[B14292589] Lee Ha Young, Kim Hye Jin, Shin Hak Sub, Han Sang Hak, Ko Seung Yeon, Song Ju Hyeon, Lee Jung Hyo, Jang Kyung Hwan, Yun Chung Weon (2014). Community structure of *Pinus
densiflora* and *Quercus
mongolica* forest in Jochimryeong to Shinbaeryeong of the Baekdudaegan. Journal of Korean Forest Society.

[B14292652] Lee Jong-Won, Yun Ho-Geun, Hwang Tae Young, An Jong-Bin (2022). Floristic inventory and distribution characteristics of vascular plants in forest wetlands of South Korea. Biodiversity Data Journal.

[B14292661] Lee Jong-Won, Yun Ho-Geun, Hwang Tae Young, Kim Kyungmin, Jung Se-Hoon, An Jong Bin (2023). Floristic inventory and distribution characteristics of algific talus slopes in a specific area of forest biodiversity in South Korea. Biodiversity Data Journal.

[B14292672] Lee Mi-Jeong, Song Hokyung (2011). Vegetation structure and ecological restoration model of *Quercus
mongolica* community. Journal of the Korean Environmental Restoration Technology.

[B14292681] Lim Seon-Mi, Kim Ji Hong (2015). The ecological characteristics of classified forest cover types in the natural forest of Sobaeksan. Journal of Forest and Environmental Science.

[B14292690] Liu Lei, Li Fengzi, Hai Long, Sa Rula, Gao Minglong, Wang Zirui, Tie Niu (2024). Priority conservation area of *Quercus
mongolica* under climate change: Application of an ensemble modeling. Sustainability.

[B14292702] Ministry of Climate Energy and Environment Enforcement rule of the Wildlife Protection and Management Act (Ordinance No. 39, p. 1). https://law.go.kr.

[B14292710] Environment Ministry of Sangtaegyegyoran saengmul jijeong gosi (Public Notice on the designation of ecosystem-disturbing species) (Ministry of Environment Notice No. 2025-165) (p. 1). Korea Legislation Research Institute. National Law Information Center (p. 1). https://www.law.go.kr/LSW//admRulLsInfoP.do?admRulId=41549&efYd=0.

[B14292734] JPL NASA (2021). NASADEM Merged DEM Global 1arc second V001. Distributed by Open Topography.

[B14292742] Science National Academy of Agricultural Deep soil texture spatial data of Korea (Simto-toseong) [Data set]. VWorld Spatial Information Download, Digital Twin National Land. Rural Development Administration. https://www.vworld.kr/dtmk/dtmk_ntads_s002.do?svcCde=MK&dsId=30493.

[B14292750] Science National Academy of Agricultural Soil drainage class spatial data of Korea (Baesu-deunggeup) [Data set]. VWorld Spatial Information Download, Digital Twin National Land. Rural Development Administration. https://www.vworld.kr/dtmk/dtmk_ntads_s002.do?svcCde=MK&dsId=30491.

[B14292758] Science National Academy of Agricultural Effective soil depth spatial data of Korea (Yuhyo-tosim) [Data set]. VWorld Spatial Information Download, Digital Twin National Land. Rural Development Administration. https://www.vworld.kr/dtmk/dtmk_ntads_s002.do?svcCde=MK&dsId=30494.

[B14292766] Resources National Institute of Biological (2021). Red data book of Republic of Korea. Vascular Plants.

[B14292774] Resources National Institute of Biological National List of Korea. https://kbr.go.kr/.

[B14292782] Resources National Institute of Biological Endemic species of Korea. https://species.nibr.go.kr/end-species?sttSn=75&pstatSn=1766.

[B14292790] Ecology National Institute of (2018). Floristic target species in Korea.

[B14292798] Ecology National Institute of (2019). Guidelines for the 5^th^ National Ecosystem Survey.

[B14292806] Ecology National Institute of List and ecological characteristics of alien species in Korea (Data set). Public Data Portal. https://www.data.go.kr/data/15012927/fileData.do.

[B14292814] Okaura Takatomi, Quang Nguyen Duc, Ubukata Masatoshi, Harada Ko (2007). Phylogeographic structure and late Quaternary population history of the Japanese oak Quercus
mongolica
var.
crispula and related species revealed by chloroplast DNA variation. Genes & Genetic Systems.

[B14292823] Park Byeong-Joo, Cheon Kwangil (2025). Herbaceous layer response to overstory vegetation changes in *Quercus
mongolica* Fisch. ex Ledeb. forests in Korea. Forests.

[B14292832] POWO Plants of the World Online. Facilitated by the Royal Botanic Gardens, Kew. https://powo.science.kew.org/.

[B14292840] Shitara Takuto, Nakamura Yukito, Krestov Pavel Vitalevich, Suzuki Shin'ichi, Hoshino Yoshinobu, Kamijo Takashi (2024). Phytosociological characteristics of Betula
davurica–Quercus
crispula forests in Japan based on a comparison with summergreen broad-leaved forests in Northeast Asia. Journal of Asia-Pacific Biodiversity.

[B14292851] Song Ju Hyeon, Yun Chung Weon (2019). Forest vegetation structure in Maruguem area from Gitdaebaegibong to Jukryeong, Baekdudaegan. Journal of Korean Society of Forest Science.

[B14292860] Suh M. H, Lee D. K (1998). Stand structure and regeneration of *Quercus
mongolica* forests in Korea. Forest Ecology and Management.

[B14292878] Um Tae Won (2014). Distribution characteristics of *Quercus
mongolica* in relation to topography and soil in Mt. Joongwang, Gangwon Province. Journal of Agriculture & Life Science.

[B14292887] Woo Jun-Hyuk, Lee Min-Ki, Chun Jung-Hwa, Lee Chang-Bae (2025). Forest strata and abiotic factors primarily regulate understory species richness rather than forest type in a temperate forest of South Korea. Biology.

[B14292896] Yu Hye Rin, You Young Han, Park Ji Won, Park Yeo Bin (2024). Structural characterization and succession prediction of the Korean Peninsula’s major deciduous broadleaf forest, *Quercus
mongolica* community. Journal of Wetlands Research.

[B14292929] Yun Joo-Wan, Jung Sung-Cheol, Koo Gyo-Sang, Lee Jung-Hyo, Yun Chung-Weon, Joo Sung-Hyun (2010). Forest vegetation classification on Sobaeksan National Park in the Baekdudaegan. Korean Journal of Environment and Ecology.

